# The relationship between leisure time physical activity patterns, Alzheimer’s disease markers and cognition

**DOI:** 10.1093/braincomms/fcae431

**Published:** 2025-01-31

**Authors:** Sarah-Naomi James, Carole H Sudre, Josephine Barnes, David M Cash, Yu-Jie Chiou, William Coath, Ashvini Keshavan, Kirsty Lu, Ian Malone, Heidi Murray-Smith, Jennifer M Nicholas, Michele Orini, Thomas Parker, Pamela Almeida-Meza, Nick C Fox, Marcus Richards, Jonathan M Schott

**Affiliations:** MRC Unit for Lifelong Health and Ageing at UCL, Department of Population Science and Experimental Medicine, University College London, London WC1E 7HB, UK; Dementia Research Centre, UCL Queen Square Institute of Neurology, University College London, London WC1N 3BG, UK; MRC Unit for Lifelong Health and Ageing at UCL, Department of Population Science and Experimental Medicine, University College London, London WC1E 7HB, UK; Dementia Research Centre, UCL Queen Square Institute of Neurology, University College London, London WC1N 3BG, UK; Centre for Medical Image Computing, University College London, London WC1V 6LJ, UK; Biomedical Computing, School of Biomedical Engineering and Imaging Sciences, King’s College London, London SE1 7EH, UK; Dementia Research Centre, UCL Queen Square Institute of Neurology, University College London, London WC1N 3BG, UK; Dementia Research Centre, UCL Queen Square Institute of Neurology, University College London, London WC1N 3BG, UK; UK Dementia Research Institute at UCL, University College London, London NW1 3BT, UK; Department of Psychiatry, Kaohsiung Chang Gung Memorial Hospital, Chang Gung University College of Medicine, Kaohsiung 833401, Taiwan; Nuffield Department of Population Health, University of Oxford, Oxford OX3 7LF, UK; Dementia Research Centre, UCL Queen Square Institute of Neurology, University College London, London WC1N 3BG, UK; Dementia Research Centre, UCL Queen Square Institute of Neurology, University College London, London WC1N 3BG, UK; Dementia Research Centre, UCL Queen Square Institute of Neurology, University College London, London WC1N 3BG, UK; Dementia Research Centre, UCL Queen Square Institute of Neurology, University College London, London WC1N 3BG, UK; Dementia Research Centre, UCL Queen Square Institute of Neurology, University College London, London WC1N 3BG, UK; Department of Medical Statistics, London School of Hygiene and Tropical Medicine, University of London, London WC1E 7HT, UK; MRC Unit for Lifelong Health and Ageing at UCL, Department of Population Science and Experimental Medicine, University College London, London WC1E 7HB, UK; Dementia Research Centre, UCL Queen Square Institute of Neurology, University College London, London WC1N 3BG, UK; UK Dementia Research Institute, Centre for Care Research and Technology, Imperial College London, London W12 0BZ, UK; Department of Medicine, Division of Brain Sciences, Imperial College London, London W12 0NN, UK; Department of Behavioural Science and Health, University College London, London WC1E 6BT, UK; Dementia Research Centre, UCL Queen Square Institute of Neurology, University College London, London WC1N 3BG, UK; MRC Unit for Lifelong Health and Ageing at UCL, Department of Population Science and Experimental Medicine, University College London, London WC1E 7HB, UK; MRC Unit for Lifelong Health and Ageing at UCL, Department of Population Science and Experimental Medicine, University College London, London WC1E 7HB, UK; Dementia Research Centre, UCL Queen Square Institute of Neurology, University College London, London WC1N 3BG, UK

**Keywords:** physical activity, Alzheimer’s disease pathology, hippocampal volume, cognitive reserve, cognitive resilience

## Abstract

We assessed the association between leisure time physical activity patterns across 30 years of adulthood with a range of *in vivo* Alzheimer’s disease-related neurodegenerative markers and cognition, and their interplay, at age 70. Participants from the 1946 British birth cohort study prospectively reported leisure time physical activity five times between ages 36 and 69 and were dichotomized into (i) not active (no participation/month) and (ii) active (participated once or more/month) and further derived into: (0) never active (not active); (1) active before 50’s only (≤43 years); (2) active from 50’s onwards only (≥53 years); (3) always active (active throughout). Participants underwent 18F-florbetapir Aβ and magnetic resonance imaging at age 70. Regression analyses were conducted to assess the direct and the moderating relationship between leisure time physical activity metrics, Alzheimer’s disease-related neurodegeneration markers (including Aβ status, hippocampal and whole-brain volume, and cortical thickness in Alzheimer’s disease signature regions) and cognition. All models were adjusted for childhood cognition, education and childhood socioeconomic position, and examined by sex. Findings drawn from 468 participants (49% female) demonstrated a direct association between being active before 50 years old (≤43 years) and throughout life (up to age 69 years), with larger hippocampal volume at age 70 (*P* < 0.05). There was little evidence that leisure time physical activity had direct effects on other brain health measures (all *P* > 0.05). However, leisure time physical activity patterns modified and attenuated the association between poorer cognitive functioning at age 70 and a range of Alzheimer’s disease-related neurodegenerative markers (Aβ status; hippocampal and whole-brain volume; cortical thickness in Alzheimer’s disease regions) (all *P* < 0.05). We found suggestive evidence that women with early markers of Alzheimer’s disease-related neurodegeneration were most sensitive to leisure time physical activity patterns: a lifetime of inactivity in women exacerbated the manifestation of early Alzheimer’s disease markers (Aβ and cortical thickness-related cognition), yet, if women were active across life or early in life, it mostly buffered these negative relationships. Engagement in leisure time physical activity in the life course is associated with better cognitive functioning at age 70, even in those with early markers of Alzheimer’s disease. If causal, this is likely via multiple pathways, potentially through the preservation of hippocampal volume, as well as via cognitive resilience pathways delaying cognitive manifestations of early markers of Alzheimer’s disease, particularly in women. Our findings warrant further research to shed light on the mechanisms of physical activity as a potential disease-modifying intervention of brain health and cognitive resilience.

## Teaser

James *et al*. find that leisure time physical activity from early adulthood is associated with larger hippocampal volume and better cognition at age 70. The study suggests that leisure time physical activity modifies the impact of Alzheimer's disease-related neurodegeneration, particularly in women, supporting its role as a potential intervention for cognitive resilience.

## Graphical Abstract

**Graphical Abstract:**
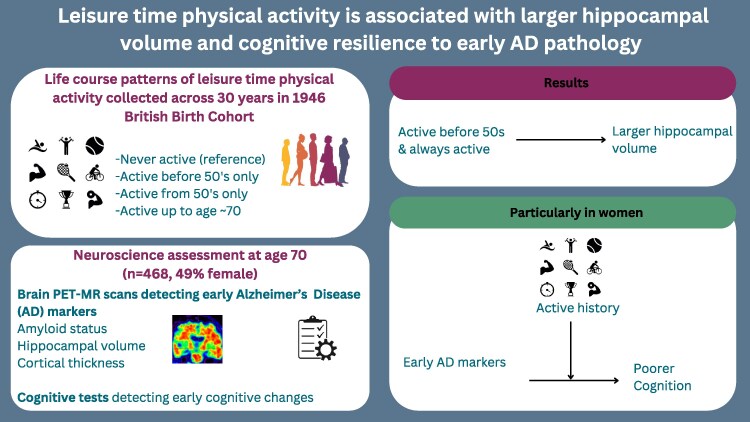

## Introduction

Extensive epidemiological studies, including meta-analyses and literature reviews, describe physical activity as a modest protective factor against Alzheimer’s disease,^1^ later-life cognitive deficits^2,3^ and cognitive decline.^4,5^ Yet, not all studies show this neuroprotective relationship^6^ and studies may be at risk of reverse directionality, where people in pre-symptomatic stages of dementia exercise less. A life course approach, following patterns of physical activity over a long period of time, can help to address this.

The cerebral pathways underlying the implicated relationship between physical activity and later-life cognition and Alzheimer’s disease are not clear.^7^ The pathways may be conferring a direct inhibitory effect on the accumulation of Alzheimer’s disease-related disease burden, which in turn may lead to better cognitive performance. For example, physical activity has been linked with reduction of Alzheimer’s disease-related pathology such as β-amyloid (Aβ)^8–11^; preservation or enhancement of hippocampal volume^11,12^; preservation of larger whole-brain volume in the face of age-related changes^13^; and a reduction in cerebrovascular pathology burden.^14^ Another possibility is that physical activity indirectly buffers the adverse and detrimental effects of Alzheimer’s disease-related neurodegeneration on cognition by enabling the maintenance of high cognitive function in the presence of Alzheimer’s disease-related pathology^15,16^ in line with the theory of cognitive resilience and reserve.^17^ In support of this, active lifestyles have been demonstrated to be associated with better cognition despite a similar level of pathology in pathology-confirmed accumulation of Alzheimer’s disease,^16,18^ in people with autosomal dominant dementia^19^ and in asymptomatic individuals.^15^ There may also be differential effects of physical activity by sex,^4^  *APOE-*ɛ4 risk status and burden of cerebral small vessel disease.^8,10,15,20,21^

Using a population-based age-homogeneous birth cohort, which has continuously followed people born in the same week of 1946, we previously demonstrated that participating in leisure time physical activity (LTPA) at any assessment in adulthood (from age 36 to 69), and to any extent (participating at least once per month), was linked with higher later-life cognitive state; but the strongest relationship was for those who engaged in physical activity for the longest, in an cumulative manner.^3^ We now extend this work, drawing on an embedded neuroscience sub-study, to assess the interrelationships between patterns of LTPA across 30 years of adulthood, and a range of *in vivo* Alzheimer’s disease-related neurodegenerative markers [Aβ burden; hippocampal volume; whole-brain volume; cortical thickness (CT) in Alzheimer’s disease-related regions] and their relationship with cognition. We investigated: (i) whether LTPA patterns are directly associated with Alzheimer’s disease-related neurodegeneration markers at age 70; (ii) whether LTPA patterns are indirectly related to brain health by moderating the relationships between Alzheimer’s disease-related neurodegeneration markers and cognition at age 70 (in line with the cognitive resilience framework^17^); (iii) whether these relationships vary by sex, APOE-ɛ4 genotype and concurrent levels of cerebral small vessel disease.

## Materials and methods

### Participants

Study participants were from Insight 46, a sub-study of the MRC National Survey of Health and Development (NSHD; the 1946 British birth cohort), which initially comprised 5362 individuals born throughout mainland Britain in 1 week in March 1946.^22^ Follow-up has included >24 contacts with the whole sample since birth. Eligibility criteria^23^ and an overview of recruitment for Insight 46^24^ are outlined elsewhere. Briefly, 502 participants aged 69–71 were assessed at University College London with a detailed clinical, cognitive and brain imaging protocol. Ethical approval for the neuroscience sub-study was granted by the National Research Ethics Service Committee London (14/LO/1173). All participants gave written informed consent. No information is provided in this manuscript that can identify any individual study member.

### Assessment of leisure time physical activity

#### Age 36, 43, 53, 60–64 and 69

As previously described,^3^ participation in LTPA was collected prospectively at ages 36, 43, 53, 60–64 and 69 years. At age 36, participation was ascertained using a modified validated Minnesota leisure time physical activity questionnaire, assessing how often people participated in a range of physical activities per month.^25^ This also assessed how often people had taken part in any sports, vigorous leisure activities or exercise in the previous month (version administered at age 43) and the previous 4 weeks (version administered at age 53, 60 and 69).^26^ Similarly to previous work in the sample, at each age, responses were categorized into: not active (no participation in LTPA/month) and active (participated in LTPA ≥1 times/month).^27–30^ Previous work in the cohort has demonstrated the consistency and similar patterns of variation between objective and self-reported instruments of physical activity.^31^

To investigate longitudinal patterns of LTPA, two periods across adulthood were defined: active before 50’s (≤43 years) and active in 50’s onwards (≥53 years). For each period, participation in LTPA ≥1 times/month was expressed in binary form (Not active and active). A LTPA categorical variable was created that represented all four possible trajectories of LTPA adulthood patterns: (0) never active (not active); (1) active before 50’s only (≤43 years); (2) active in 50’s onwards (≥53 years); (3) always active (active before and after 50’s).

### Alzheimer’s disease-related neurodegeneration markers at age 69–71

Neuroimaging was performed on a single Siemens Biograph mMR 3T PET/MRI scanner (Siemens Healthcare, Erlangen), with simultaneous acquisition of dynamic PET data from 0 to 60 min post-injection of 370 MBq ^18^F-florbetapir (Amyvid) and MR sequences including volumetric (1.1 mm isotropic) T1 and fluid-attenuated inversion recovery (FLAIR). The full imaging protocol has been described previously.^23^

#### PET amyloidβ (Aβ)

As previously described,^32^ global standardized uptake value ratios (SUVRs) were calculated from a composite of cortical regions of interest, normalized to eroded subcortical white matter. Aβ status (+/−) was determined using Gaussian mixture modelling, taking the 99th percentile of the lower (Aβ−) Gaussian as the cut-point (0.6104), whereby Aβ+ indicates greater Aβ load.

#### Brain and hippocampal volume

Volumetric T1-weighted and FLAIR images underwent visual QC, before processing using automated pipelines^23^: whole-brain segmentation using multi-atlas propagation and segmentation^33^ and hippocampal volume using similarity and truth estimation for propagated segmentations^34^ with appropriate manual editing. Models including hippocampal aandd brain volume were all adjusted for total intracranial volume (TIV), calculated using statistical parametric mapping 12.

#### Cortical thickness in Alzheimer’s disease-region

Cortical thickness estimation was performed using FreeSurfer version 6.0. A cortical thinning signature for Alzheimer’s disease was derived using the Mayo Alzheimer’s disease signature, comprising entorhinal, fusiform, inferior and middle temporal cortical regions.^35,36^

### Cognitive measures at age 69–71

Participants undertook an adapted version of the pre-clinical Alzheimer cognitive composite (PACC), composed of four tests: the Mini Mental State Examination, Logical Memory IIa from the Wechsler Memory Scale-Revised, Digit-Symbol Substitution test from the Wechsler Adult Intelligence Scale-Revised and the 12-item Face-Name test (FNAME-12).^37^ Each test was normalized to the analytical Insight 46 sample. The PACC is designed to track sensitive cognitive decline in the preclinical phase of Alzheimer’s disease. Individuals were categorized as APOE-ɛ4 carriers or non-carriers.

### Covariables

Earlier life covariables were childhood cognition,^38^ childhood socioeconomic position (SEP)^39^ and educational attainment.^40^ The standardized sum of four tests of verbal and nonverbal ability at age eight represented childhood cognition. Childhood SEP was recorded from paternal occupation according to the Registrar General’s classification of the paternal occupation^41^ and dichotomized into ‘Unskilled, partly skilled or skilled manual’ and ‘Skilled non-manual, intermediate or professional’. Education up to age 26 was categorized into three groups based on the Burnham Scale^42^: ‘None attempted’; ‘Vocational or ordinary (O’ level or equivalent)’ and ‘advanced (A-Level) or higher education’. A validated, unsupervised, automated algorithm, Bayesian Model Selection (BaMoS),^43^ was used to segment white matter hyperintensities jointly from 3D T1 and FLAIR images, followed by visual QC, generating a global White matter hyperintensity volume (WMHV) excluding infratentorial regions. Higher WMHV indicates worse small vessel disease.

### Statistical analysis

#### Sample

Participants were included in this analysis if they had at least one measure of physical activity across adulthood and at least one cognition and Alzheimer’s disease-related neurodegeneration marker at age 70 (Aβ status; hippocampal volume; whole-brain volume; CT in Alzheimer’s disease-related regions). The *t*-tests and χ^2^ tests were conducted to assess sex differences in the descriptive characteristics. All statistical analyses were calculated utilizing STATA V.18 (STATA Corp, TX, USA) with a 0.05 significance level. Supplementary Fig. 1 shows a flow chart of the analytical Insight 46 neuroimaging sub-study sample and provides an overview of the recruitment procedure. We additionally compared key characteristics of people still active in the NSHD sample at age 69 with those in Insight 46 (Supplementary Table 1). As previously reported,^44^ those in Insight 46 were more highly educated than the wider NSHD sample (54% versus 39% were in education over the age of 17), and the analytical sample had slightly higher childhood cognition scores (0.4 versus 0.1), Insight 46 participants also had higher rates of engagement in LTPA at every age (e.g. at age 69, 54% versus 39% were physically active). However, the analytical neuroimaging sub-study and wider NSHD sample had a comparable sex ratio (50%), comparable cognitive scores on a measure of cognitive state at age 69 (93/100 versus 92/100) and comparable rates of APOE-e4 carriage (30%).

#### Analytical approach

**Figure 1:**
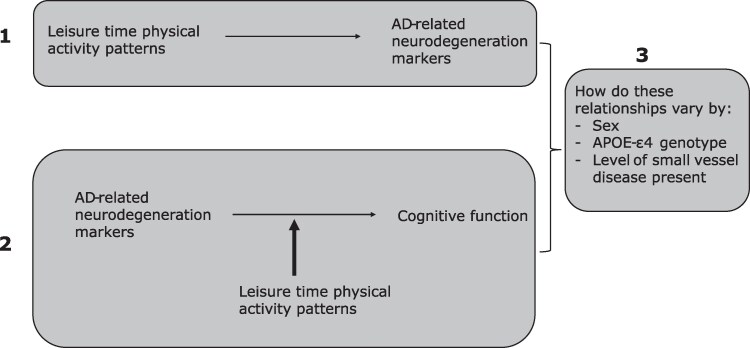
**An illustration of research questions.** The aims were to (1) investigate whether leisure time physical activity patterns are directly associated with Alzheimer’s disease (AD)-related neurodegeneration markers at age 70 (Aβ status, hippocampal volume, total brain volume and cortical thickness in Alzheimer’s disease regions); (2) investigate whether leisure time physical activity patterns are indirectly related to brain health by moderating the relationship between Alzheimer’s disease-related neurodegeneration markers and cognition at age 70 (in line with the cognitive resilience framework) and (3) investigate whether these relationships vary by sex, APOE-ɛ4 genotype and concurrent levels of cerebral small vessel disease.

Figure 1 shows an illustrative diagram of the aims.

We use the term ‘interaction’ to describe the joint effect of two exposures on an outcome (e.g. an interaction test between LTPA and amyloid status on cognition will indicate whether there is a combined effect of LTPA and amyloid on cognition that is different than the sum of their separate effects).^45^ We use the term ‘effect modification’ to indicate whether the relationship between an exposure(s) and outcome differs across population subgroups, performed by stratifying the population and comparing the marginal effects in the subgroups (e.g. does the relationship between LTPA and amyloid status on cognition differ in men and women?).^45^

#### The direct relationship between LTPA patterns and Alzheimer’s disease-related neurodegeneration

Multivariable regression models were conducted to assess the association between LTPA patterns and Alzheimer’s disease-related neurodegeneration measures at age 70. Linear regression models were conducted for brain volume, hippocampal volume (used as continuous variables and standardized to the analytical sample after adjustment for TIV) and CT-Alzheimer’s disease. Logistic regression models were used for Aβ status (used as binary variable using Aβ− as the reference). LTPA was used as an indicator variable with (i) never active as the reference group versus (ii) those who were active (participated in LTPA ≥1 times per month) before 50’s only (age ≤ 43); (iii) those who were active from 50’s onwards only (age ≥53) and (iv) those who were always active throughout adulthood. All models adjusted for sex, scan age, childhood cognition, childhood socioeconomic position and education. Adjustments were not made for multiple comparisons in line with previous studies.^46^

To investigate how manual work and poor health may confound some of these relationships, we re-ran the main analyses adjusting for manual work status (up to age 60), and history of poor mental health and a number of poor physical health indices up to age 69, including self-rated poor general health and longstanding illness, chronic pain and pain that severely limits daily activity (more detail provided in Supplementary material II).

#### The indirect relationship: does the relationship between markers of Alzheimer’s disease-related neurodegeneration and cognition differ by LTPA patterns?

To investigate whether the relationship between markers of Alzheimer’s disease-related neurodegeneration and cognition differ by LTPA patterns, in line with the cognitive resilience framework, we first ran linear regression models which assessed the overall direct relationship between each imaging metrics of interest at age 70 (Aβ status; hippocampal volume; brain volume; CT-Alzheimer’s disease) and cognition. We then added an additional two-way interaction term between LTPA patterns and each imaging metric of interest to assess whether the LTPA patterns had any effect in varying the relationship between brain health and cognition. The overall interaction by LTPA was ascertained by conducting a post-estimation Wald test (taking *P* < 0.10 as evidence of an interaction). Post-estimation marginal effects of the associations for each LTPA group were additionally obtained. *Post hoc* differences in how the relationships differed between LTPA groups, using the inactive group as the reference group, were ascertained using marginal contrasts. All models adjusted for sex, scan age, childhood cognition, childhood socioeconomic position and education. We re-ran these main analyses adjusting for adult manual work and poor general health (see Supplementary for more detail).

#### Do these relationships vary by sex, APOE-e4 and cerebral small vessel disease?

All main analyses were re-run to assess if there was a differential influence of sex (male or female), APOE-ɛ4 status (carrier or non-carrier) and WMHV as a marker of cerebral small vessel disease [for ease of interpretation, WMHV was split into a categorical variable of three equally sized tertiles: lowest WMHV (0), average WMHV (1) and highest WMHV (2)]. This approach was conducted by adding two-way (i.e. sex*LTPA) or three-way (i.e. sex*LTPA*Aβ) interactions to the models previously described. Differences in how the relationships differed by these characteristics were ascertained using marginal contrasts.

## Results

Study characteristics of the Insight 46 sub-sample (Max *n* = 468, 49% female) are shown in Table 1. Generally, rates of LTPA decreased with age, from 73% physically active at age 36, to 54% being physically active at age 69.

**Table 1:** Characteristics of the analytical sample

	*n*	Units	All	Men (*n* = 241)	Women (*n* = 230)	*P*-value for sex difference*
Demographics						
Sex	471	Female (*n*, %)	230 (49%)			
APOE-ɛ4	438	APOE ɛ4 non-carriers (*n*,%)	308 (70%)	154 (68%)	154 (72%)	0.4
		APOE ɛ4 carriers (*n*, %)	130 (30%)	71 (32%)	59 (28%)	
Childhood cognition	471	Standardized (SD)	0.4 (0.7)	0.37 (0.8)	0.43 (0.7)	0.3
Childhood social class	467	Manual (*n*, %)	198 (42%)	95 (40%)	103 (46%)	0.2
		Non-manual (*n*, %)	269 (58%)	146 (60%)	123 (54%)	
Educational attainment	471	No qualifications (*n*, %)	74 (16%)	36 (15%)	38 (17%)	<0.01
		Up to age 16 (*n*, %)	142 (30%)	59 (24%)	83 (36%)	
		Up to age 17 or higher (*n*, %)	255 (54%)	146 (61%)	109 (47%)	
Leisure time physical activity (LTPA) patterns						
Active at age 36	434	None (*n*, %)	118 (27%)	56 (25%)	62 (30%)	0.4
		Active ≥1times/month (*n*, %)	316 (73%)	165 (75%)	151 (70%)	
Active at age 43	454	None (*n*, %)	176 (39%)	84 (36%)	92 (42%)	0.2
		Active ≥1times/month (*n*, %)	278 (61%)	149 (64%)	129 (58%)	
Active at age 53	463	None (*n*, %)	151 (33%)	76 (32%)	75 (33%)	0.8
		Active ≥1times/month (*n*, %)	312 (67%)	159 (68%)	153 (67%)	
Active at age 60	466	None (*n*, %)	240 (52%)	129 (43%)	111 (49%)	0.3
		Active ≥1 times/month (*n*, %)	226 (48%)	110 (47%)	116 (51%)	
Active at age 69	464	None (*n*, %)	212 (46%)	113 (47%)	99 (44%)	0.3
		Active ≥1 times/month (*n*, %)	252 (54%)	124 (53%)	128 (56%)	
Life course patterns of LTPA (≥1 times per month) across different time periods	464	Never active (*n*, %)	30 (7%)	16 (7%)	14 (6%)	0.7
		Active before 50’s only (≤43 years) (*n*, %)	54 (12%)	30 (13%)	24 (11%)	
		Active from 50’s onwards only (≥53 years) (*n*, %)	51 (11%)	23 (10%)	28 (12%)	
		Always active (*n*, %)	329 (70%)	169 (70%)	160 (71%)	
Cognitive function, age 70						
PACC score	471	Standardized mean (SD)	0 (0.73)	−0.2 (0.7)	0.2 (0.7)	<0.01
Brain health measures, age 70						
Age at scan	471	Mean (SD) years	70.7 (0.7)	70.7 (0.7)	70.7 (0.7)	0.8
Aβ status	459	Positive (*n*, %)	86 (19%)	47 (20%)	39 (17%)	0.4
Aβ standardized uptake value ratio	462	Mean (SD)	1.1 (0.2)	0.6 (0.1)	0.6 (0.1)	0.3
Hippocampal volume	468	Mean (SD) mL	3.1 (0.3)	3.3 (0.3)	3.0 (0.3)	<0.01
TIV-adjusted hippocampal volume	468	Mean (SD) mL	0.01 (0.3)	0.02 (0.3)	−0.01 (0.3)	0.3
Brain volume	468	Mean (SD) mL	1101.1 (99)	1152.7 (87)	1047.3 (82.5)	<0.01
TIV-adjusted brain volume	468	Mean (SD) mL	0.01 (45)	−6.0 (45)	6.2 (45)	0.01
TIV	468	Mean (SD) mL	1433.3 (133)	1519.6 (105.9)	1343.2 (92.47)	<0.01
CT, Alzheimer’s disease regions	468	Mean (SD) mm	2.8 (0.1)	2.9 (0.1)	2.8 (0.1)	0.3

LTPA, leisure time physical activity; SD, standard deviation; APOE-e4, apolipoprotein E; PACC, preclinical Alzheimer’s cognitive composite; TIV, total intercranial volume.

*Indicates the *P* value from *t*-tests (continuous variables) and *x*^2^ tests (categorical variables) that were conducted to assess sex differences within these characteristics.

### What is the direct relationship between LTPA patterns and Alzheimer’s disease-related neurodegeneration?

**Figure 2:**
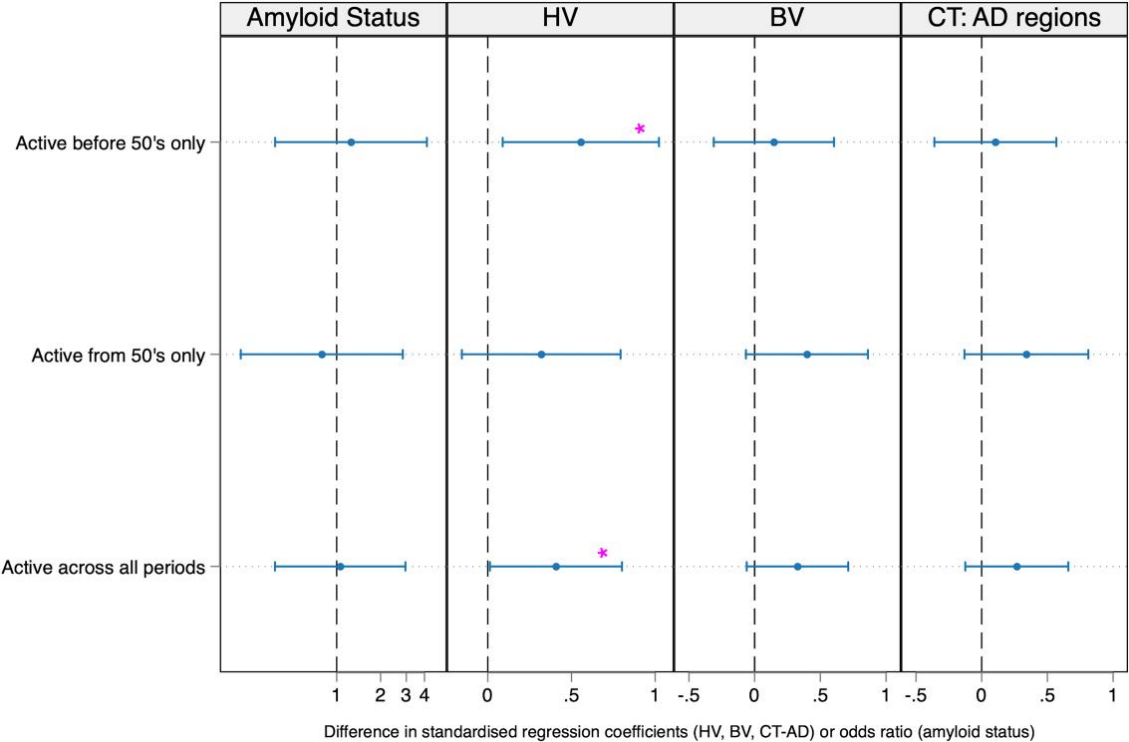
**Direct associations between leisure time physical activity (LTPA) patterns and Alzheimer’s disease-related neurodegeneration markers at age 70.** Odds ratio with 95% confidence intervals are presented from logistic regression (Aβ status) and standardized coefficients and 95% confidence intervals are presented from linear regression models (HV, hippocampal volume; BV, total brain volume; CT-Alzheimer’s disease, cortical thickness in Alzheimer’s disease regions; *n* = 464). ‘Active’ is considered participating in LTPA ≥1 times per month. Regression models demonstrated that, compared to those who were never active across adulthood, those who were active before 50’s only (age ≤ 43), and those who were always active, had higher hippocampal volume (*b* = 0.6, *P* = 0.02; *b* = 0.4, *P* = 0.03, respectively). All other coefficients were *P* > 0.05 and are reported in Supplementary Table 2. All models adjusted for sex, scan age, childhood cognition, childhood social class, education and TIV (for HV and BV).

Compared to those who never participated in LTPA across adulthood, those who were active before 50’s only; and throughout life; had a significantly larger hippocampal volume at age 70, after adjustment for TIV, sex, scan age, childhood socioeconomic position, childhood cognition and education (Fig. 2, Supplementary Table 2). For example, those who were active before 50 had a mean 0.6 SD (95% CI 0.09–1.02) larger hippocampal volume than those who were never active, equivalent to 6% or 0.18 mL larger volume. Those who were active in their 50’s onwards showed a similar pattern of larger hippocampal volume, but this was not statistically significant (*P* = 0.19). There was limited evidence of a direct association between LTPA patterns and lower Aβ status, larger brain volume or CT-Alzheimer’s disease, although the direction of associations were in the expected direction.

There was no evidence of modification by sex, APOE-e4 or WMHV (Supplementary Table 2). The relationships between LTPA and hippocampal volume were similar when manual work was incorporated into the models and was strengthened with adjustment for history of poor mental or physical health (Supplementary Fig. 2).

### Does the relationship between Alzheimer’s disease-related neurodegeneration and cognition differ by LTPA patterns?

**Figure 3:**
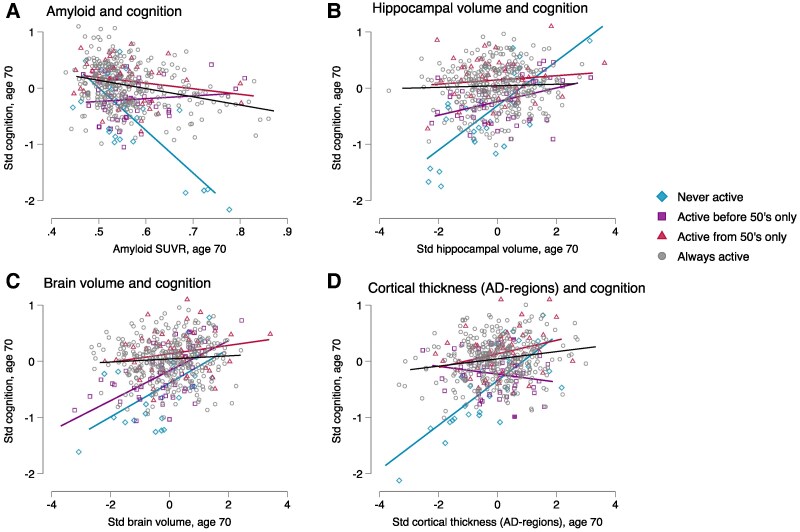
**Plots modelling the relationship between Alzheimer’s disease-related neurodegeneration measures and cognition, by life course leisure time physical activity (LTPA) patterns.** The data points represent predicted cognitive function at age 70 plotted against each imaging metric of interest, by LTPA, derived from linear regression models modelling the relationship between the standardised imaging metric of interest on cognition, adjusting for sex, scan age, childhood cognition, childhood socioeconomic position, education and with interaction terms between LTPA patterns and the imaging metric of interest. LTPA patterns include those who were (i) never active across adulthood; those who were active (participated in LTPA ≥1 times per month) in (ii) before 50’s only (age ≤ 43); (iii) from 50’s onwards only (age ≥53) and (iv) always active throughout adulthood (total *n* = 464). Wald tests revealed significant LTPA-by-imaging metric interactions for the relationship between (**A**) amyloid and cognition (*P* < 0.01), (**B**) hippocampal volume and cognition (*P* < 0.01), (**C**) brain volume and cognition (*P* < 0.01) and (**D**) CT in Alzheimer’s disease-regions and cognition (*P* = 0.03). STD, standardized; SUVR, global standardized uptake value ratios.

The relationship between every Alzheimer’s disease-related neurodegeneration marker and cognition at age 70 differed by life course LTPA activity patterns after adjustment for sex, scan age, childhood socioeconomic position, childhood cognition and education (all interactions *P* < 0.05; Table 2, Fig. 3).

**Table 2:** The relationship between brain health measures and cognition function at age 70 (preclinical Alzheimer’s cognitive composite, PACC), by life course leisure time physical activity (LTPA) patterns

	Overall interaction between LTPA*brain health	Difference in coefficients versus reference group (not active)	Relationship between brain health and cognition			Three-way interaction with sex	Three-way interaction with APOE-e4	Three-way interaction with WMHV
	*P*-value	*P*-value	*ß*	*P*	CI 95%	*P*-value	*P*-value	*P*-value
**Aβ status and PACC**			**−0**.**25**	**<0**.**01**	**−0.40,−0.10**			
By LTPA patterns:								
Never active	**<0**.**01**	Reference	**−1**.**67**	**<0**.**01**	**−2.27,−1.07**	**<0**.**01**	0.32	0.21
Active before 50’s only (≤43 years)		*P* < 0.01	−0.04	0.85	−0.45,0.37			
Active from 50’s onwards only (≥53 years)		*P* < 0.01	−0.34	0.17	−0.84,0.15			
Always active		*P* < 0.01	−0.17	0.21	−0.34,0.10			
**Hippocampal volume and PACC**			**0**.**06**	**0**.**03**	**0.01,0.12**			
By LTPA patterns:								
Never active	**<0**.**01**	Reference	**0**.**44**	**<0**.**01**	**0.26,0.62**	0.85	0.89	0.13
Active before 50’s only (≤43 years)		*P* < 0.01	0.09	0.25	−0.06,0.25			
Active from 50’s onwards only (≥53 years)		*P* < 0.01	0.03	0.69	−0.13,0.20			
Always active		*P* < 0.01	0.01	0.91	−0.07,0.08			
**Brain volume and PACC**			**0**.**07**	**0**.**02**	**0.01,0.13**			
By LTPA patterns:								
Never active	**<0**.**01**	Reference	**0**.**27**	**<0**.**01**	**0.07,0.46**	0.25	0.99	0.89
Active before 50’s only (≤43 years)		0.4	**0**.**26**	**<0**.**01**	**0.10,0.43**			
Active from 50’s onwards only (≥53 years)		0.1	0.10	0.25	−0.07,0.27			
Always active		*P* < 0.01	−0.01	0.85	−0.08,0.06			
**Cortical thickness Alzheimer’s disease-regions and PACC**			**0.10**	**<0**.**01**	**0.04,0.16**			
By LTPA patterns:								
Never active	**0**.**03**	Reference	**0**.**31**	**<0**.**01**	**0.13,0.50**	**<0**.**01**	0.16	0.15
Active before 50’s only (≤43 years)		0.9	0.06	0.55	−0.13,0.25			
Active from 50’s onwards only (≥53 years)		0.2	0.16	0.09	−0.02,0.34			
Always active		*P* < 0.01	0.06	0.11	−0.01,0.13			

Coefficients and 95% confidence intervals are presented from linear regression models which assess the relationship between brain health (Aβ status; standardized TIV-adjusted hippocampal volume; standardized TIV-adjusted total brain volume; standardized CT in Alzheimer’s disease regions) and cognition at age 70 (preclinical Alzheimer’s cognitive composite, PACC). The ‘overall interaction between LTPA*brain health’ column indicates the *P*-value for interactions assessing the overall effect between LTPA patterns and brain health on PACC scores. ‘Difference in coefficients versus reference group (not active)’ column provides the *P*-value of *post hoc* tests that indicate how the relationship between brain health and PACC differs by LTPA groups compared to the inactive group. ‘Relationship between brain health and cognition’ column indicates the relationship between brain health and PACC within each LTPA group (i) never active across adulthood; those who were active [participated in LTPA ≥1 times per month) in (ii) before 50’s only (age ≤ 43); (iii) from 50’s onwards only (age ≥53) and (iv) always active throughout adulthood]. All models adjusted for sex, scan age, childhood cognition, childhood socioeconomic position, education and TIV (for hippocampal and brain volume). Three-way interaction test for how these modifying relationships vary by sex (dichotomous), APOE-e4 carrier status (dichotomous) and WMHV as three tertiles (ordinal). Bold text denotes significance at *P* < 0.05.

LTPA, leisure time physical activity; APOE-e4, apolipoprotein E; PACC, preclinical Alzheimer’s cognitive composite; TIV, total intercranial volume; WMHV, white matter hyperintensity volume.

For example, the relationship between Aβ positivity and poorer cognitive function was strongest in those who were never active in adulthood (*b* = −1.67, *P* < 0.01), but the relationship was attenuated in those who were active before 50’s only (−0.04, *P* = 0.85), from 50’s onwards only (−0.34, *P* = 0.17), or throughout life (−0.17, *P* = 0.12) (Table 2, Fig. 3A).

A similar pattern emerged for other Alzheimer’s disease-related neurodegeneration measures including hippocampal volume (Fig. 3B); brain volume (Fig. 3C); and CT-Alzheimer’s disease regions (Fig. 3D); generally, the relationship between poorer Alzheimer’s disease-related neurodegeneration markers and poorer cognitive function was strongest in those who were never active but was attenuated in those who were active (Table 2 and Fig. 3).

Results remained largely similar with adjustment for manual work (Supplementary Table 3). The relationship between brain health (amyloid status, hippocampal volume and CT-Alzheimer’s disease) with cognition in the inactive group strengthened with adjustment for poor mental or physical health (Supplementary Table 4). For example, the relationship between CT-Alzheimer’s disease and cognition in the base model was *b* = 0.31, *P* < 0.01 and it increased to 0.56, *P* < 0.01 with adjustments.

#### Modification by characteristics

**Figure 4:**
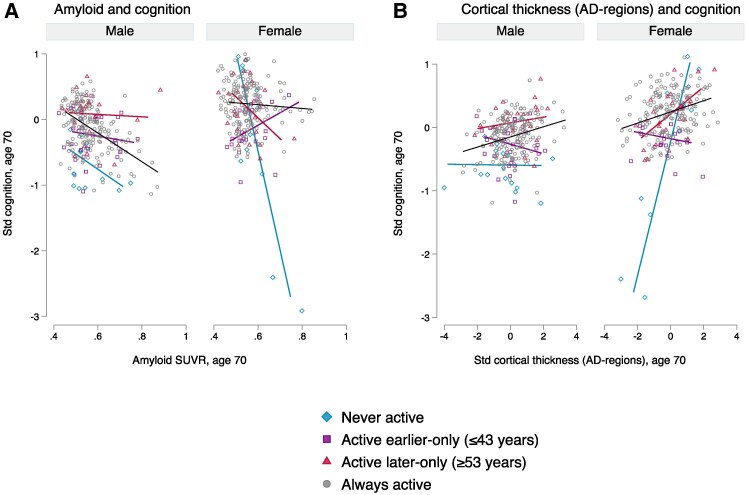
**Sex-stratified plots modelling the relationship between (A) aβ and cognition and (B) Cortical thickness in Alzheimer’s disease regions and cognition, by leisure time physical activity (LTPA) patterns.** The data points represent predicted cognitive function at age 70 plotted against each imaging metric of interest, by LTPA and sex, derived from linear regression models assessing the relationship between the imaging metric of interest on cognition, adjusting for scan age, childhood cognition, childhood socioeconomic position, education and with interaction terms between sex, LTPA patterns and the imaging metric of interest (*n* = 464). LTPA patterns include those who were (i) never active across adulthood; those who were active (participated in LTPA ≥1 times per month) in (ii) before 50’s only (age ≤ 43); (iii) from 50’s onwards only (age ≥53) and (iv) always active throughout adulthood. Wald tests revealed significant sex-by-LTPA-by-imaging metric interactions for (**A**) amyloid and cognition (*P* < 0.01) and (**B**) CT in Alzheimer’s disease regions and cognition (*P* < 0.01). STD, standardized; SUVR, global standardized uptake value ratios.

Sex modified the relationship between LTPA and Aβ positivity on cognition (Fig. 4A), and between LTPA and CT-Alzheimer’s disease regions on cognition (Fig. 4B); these relationships seemed to be more sensitive in women (both *P* < 0.01, Table 2, Supplementary Table 5). For example, the relationship between Aβ positivity and poorer cognition was strongest in women who were never active (−2.49, *P* < 0.01). This relationship attenuated when two women with the poorest cognitive function (and Aβ positive) were excluded from the analysis (−3 SD in PACC: *b* = −0.96, *P* < 0.05, Supplementary Fig. 3), suggesting that this relationship is largely driven by inactive women who likely already have early stages of Alzheimer’s disease.

## Discussion

### Main findings

In a population-based cohort of people born in the same week and followed since birth, we demonstrate that engagement in LTPA before age 50 (≤43 years) and throughout life (up to age 69 years) was directly associated with larger hippocampal volume at age 70, but was not directly associated with other early Alzheimer’s disease-related neurodegeneration measures (Aβ status; CT in Alzheimer’s disease-related regions). Second, we found that being engagement in LTPA buffers the early cognitive manifestations of a range of early Alzheimer’s disease-related brain neurodegeneration measures (Aβ status; hippocampal volume; whole-brain volume and CT in Alzheimer’s disease-related regions). This finding supports the notion that LTPA contributes to cognitive resilience and reserve, slowing the Alzheimer’s disease continuum. Interestingly, women with early markers of Alzheimer’s disease-related neurodegeneration were most sensitive to LTPA patterns: a lifetime of inactivity in women exacerbated the manifestation of early Alzheimer’s disease markers (Aβ-related cognition; CT-Alzheimer’s disease-related cognition); yet, if women were active, to any extent throughout life, it mainly buffered these negative relationships. If causal, our findings emphasize the importance of encouraging people to engage in leisure time physical activity, at any time in the life course, and in turn this may confer beneficial effects to brain health via multiple pathways, including the preservation of hippocampal volume, and via cognitive resilience pathways which delays cognitive manifestations of early markers of Alzheimer’s disease, particularly in women.^47,48^

### A direct relationship between leisure time physical activity and hippocampal volume

Being active was associated with larger hippocampal volume at age 70. Notably, the strongest relationship was for those who were active before 50 (≤43 years); and then for those who were active throughout life (up to age 69 years); and then for those who were active only from their 50’s onwards, which showed a similar, but not significant, direction of effects. The strongest association, demonstrated in those who were active before 50, is interesting and may reflect a presumed greater intensity or duration of exercise at this younger age, although this presumption is speculative and is an active area of research in our group. Interestingly, the association between LTPA and hippocampal volume strengthened in all groups when poor physical or mental health was adjusted, suggesting that poor health was masking some of the underlying relationships between physical activity and hippocampal volume.

A range of intervention studies have demonstrated associations between physical activity and larger hippocampal volume and preservation of age-related hippocampal atrophy,^47,49^ including a 24-month study in sedentary older adults, which found adherence to the multi-modal intervention of moderate physical activity was associated with larger hippocampal volume.^50^ Exploring the potential mechanisms that could directly affect hippocampal volume, such as increasing hippocampal perfusion, increasing synaptic plasticity and connectivity and neuronal density, are warranted.^47^

### LTPA is associated with sustained cognitive function, even in those with early Alzheimer’s disease pathology

As previously shown in this cohort^3^ and others^4^ participating in LTPA across adulthood was linked with higher later-life cognition. We now expand on this and demonstrate that participating in LTPA is associated with sustained cognitive function, even in those with early *in vivo* markers of Alzheimer’s disease-related neurodegeneration. Our results strongly support the notion that LTPA across the life course can contribute to cognitive reserve and resilience later in life.^16,17^ Cognitive reserve is defined as the dynamic adaptability to sustain cognitive function in the face of brain ageing, pathology or insults.^17^ This work builds on crucial and ongoing evidence in the same cohort that alongside education and occupation, engagement in LTPA can be embedded into a proxy of factors that contribute to differences in cognitive reserve.^51^

Very few studies have been able to address the modifying effect of life course LTPA patterns on early *in vivo* markers Alzheimer’s disease-related pathology and cognition. However, our findings are in line with a Harvard Ageing Brain Study (*n* = 182) of asymptomatic older adults, which found that greater baseline physical activity, assessed using an objective pedometer, was related to slower PET Aβ-related cognitive decline and PET Aβ-related volume loss,^15^ independently of vascular risk. Another study based in the at-risk Wisconsin registry for Alzheimer’s prevention cohort (*n* = 69) showed that greater baseline cardiorespiratory fitness was related to better CSF Aβ-related cognition.^52^ Active lifestyles and increases in physical activity have also been associated with better cognition despite a similar level of pathology-confirmed accumulation of Alzheimer’s disease and other dementia-related pathologies^16,17^; and in people with autosomal dementia.^19^ We add to this body of work by assessing a range of early *in vivo* biomarkers of Alzheimer’s disease neurodegeneration and disentangle life course LTPA patterns that may be more important in conferring this buffering effect of Alzheimer’s disease-related pathology (i.e. do you see the relationship only in those who are always active, or also in those who were only active earlier or later in life). Notably, our findings did not show substantial differences between timing of LTPA for effect modification of Aβ, but instead demonstrated that being active, at any time across adulthood, could help buffer the detrimental effects of Aβ-related cognition and hippocampal volume at age 70, compared to those who were never active. Together our findings suggest that being active may buffer the early subtle cognitive manifestations of Aβ. This warrants further research to delineate the type, frequency and timing of physical activity that can help sustain cognition and brain volume changes in the face of early Aβ pathology, for as long as possible.

Notably, the relationship between smaller brain volume and poorer cognition were similar for those who were never active and those who were active before 50’s only. Given that those who were active before 50’s showed a buffering effect for other brain health metrics, this finding could reflect differential pathways that mitigate smaller brain volume-associated cognition that is not linked to physical activity earlier in adulthood (e.g. through brain structure reserve mechanisms). In contrast, there may be pathways linked to physical activity earlier in adulthood that are related to more specific Alzheimer’s disease-related pathology (e.g. through compensatory mechanisms, neurogenesis, etc.). Further work is needed to explore these differential aspects of cognitive and brain resilience.

### Sex differences in susceptibility to effect modification

Interestingly, we find evidence that women may be more sensitive to LTPA patterns: inactivity in women exacerbated cognitive functioning associated with Aβ deposition and CT in Alzheimer’s disease-regions; largely driven by inactive women already on the Alzheimer’s disease continuum. In men, the modifying effect of LTPA patterns on these relationships was less prominent. This suggests that Aβ-related changes may be exacerbated in inactive women. Given that women are more likely to develop Alzheimer’s disease, our findings strongly support the encouragement of physical activity across life in women and emphasise the importance of looking at sex differences within studies interested in Alzheimer’s disease^53^. Our findings warrant urgent investigation to better understand why inactive women may be more susceptible to earlier cognitive manifestations of Alzheimer’s disease pathology,^4^ and to understand causes of inactivity with age.

### Lack of a direct relationship between LTPA and Aβ

We were not able to provide evidence of the direct association between physical activity and Aβ burden. While animal models provide the best evidence that physical activity may directly impact Aβ deposition,^54^ in humans, results using Aβ biomarkers (brain, CSF and blood) are more inconsistent. A recent meta-analysis and review of physical activity and Aβ studies reported while the number of studies were limited, overall there is a non-significant association between physical activity with brain and blood Aβ.^55^ However, very few studies address the modifying effect of physical activity on Aβ-related cognition or Aβ-related brain volume changes, which could elucidate whether interventions buffer or delay the toxic and downstream effects of Aβ. Consideration of durations and types of physical activity may also be important.

### Cerebral small vessel disease

We did not find evidence that having high or low levels of cerebral small vessel disease (as indexed by WMHV) moderated the interrelationship between physical activity, Alzheimer’s disease-related neurodegeneration and cognition. Studies investigating the relationship between physical activity and WMHV in cognitively normal older sample are inconsistent,^14^ with some suggesting that engagement in activity can help diminish age-related WMHV load,^56^ whereas others show null results, which may result from differences in WMHV definitions and derivation.^57^ However, recent moderate-intensity training interventions of 24 months^58^ and 5 years,^59^ found no effect on WMHV volume and WMH change in older adults. Age is a key factor for WMHV burden but the distribution of WMHV is fairly low in this sample at this age; it will be important to consider the interrelationships between physical activity Alzheimer’s disease-related neurodegeneration and WMHV changes as the cohort ages and expected pathology increases.

### Strengths and limitations

The sample is based on the longest continuously running birth cohort, which has multiple prospective measures of physical activity spanning over 30 years. This enabled us to tease apart potential important effects of patterns LTPA in the life course. Other strengths include the embedded range of *in vivo* Alzheimer’s disease-related neurodegeneration indices such as Aβ, hippocampal volume and CT.

However, it is also important to consider the role of other dementia-related pathways that may be involved in conferring beneficial physical activity effects. For example, animal studies have indicated a role of tau pathways, increased clearance of toxins and microglial activation through glymphatic systems, changes to cerebral blood flow and cerebrovascular health, glucose and Aβ metabolism, maintenance of motor networks, neuroinflammation and regulation in neuromodulators and neurotrophic factors.^47,60–62^

Similar to all longitudinal studies, there was a disproportional attrition of participants who were disadvantaged, regarding lower SEP, education attainment and childhood cognition,^24,63^ which was even more selected in the neuroimaging sub-sample. Second, data on physical activity in this study is fairly crude and only represents self-reported leisure-time physical activity, not occupational physical activity, or PA involved in housekeeping and other physical activities. It also does not take into account objective measures of physical activity, cardiorespiratory fitness^64^ exercise adherence, exercise intensity or duration or associated lifestyle factors^65^ or effects of resistance exercises, which may show the most beneficial effects on cognition.^48^

Continued follow-up in the cohort, as some individuals in the cohort will go on to develop Alzheimer’s disease-related symptoms, will be invaluable in providing insight into whether physical activity confers effects directly on progression of disease markers, and whether physical activity buffers effects of cognitive deterioration in the presence of disease markers that cause dementia, ultimately delaying dementia onset, in line with theories of cognitive reserve.^66^

## Conclusion

Our findings suggest that engaging in LTPA is associated with larger hippocampal volumes and better cognitive functioning at age 70, even in individuals with early Alzheimer’s disease biomarkers. Women seemed to have a greater sensitivity to physical inactivity. If replicated, our findings emphasize the importance of promoting LTPA at all life stages, and in turn this may confer beneficial effects to brain health via multiple pathways, including the preservation of hippocampal volume,^50^ as well as building brain and cognitive resilience^17^ to delay cognitive manifestations of early markers of Alzheimer’s disease. Public health policies should emphasize creating supportive environments and educating the public on the cognitive benefits of lifelong physical activity to potentially reduce the symptomatic burden of Alzheimer’s disease. Our findings warrant further research to shed light on the mechanisms of physical activity as a potential disease-modifying intervention to delay the impairing symptoms of dementia.

## Supplementary Material

fcae431_Supplementary_Data

## Data Availability

Data used in this publication are available to bona fide researchers upon request to the NSHD Data Sharing Committee via a standard application procedure. Further details can be found at http://www.nshd.mrc.ac.uk/data. doi: 10.5522/NSHD/Q102; 10.5522/NSHD/Q103.
