# Supplementary material for: The relationship between leisure time physical activity patterns, Alzheimer’s disease markers and cognition
Source: Brain Commun. 2025 Jan 31;7(1):fcae431. doi: 10.1093/braincomms/fcae431 (PMC11781833; doi:10.1093/braincomms/fcae431)
Supplement: fcae431_Supplementary_Data [file fcae431_supplementary_data.pdf]

Supplementary Materials

Supplementary material I: Sample detail

Supplementary Figure 1[1]: Deriving the sample for the neuroscience sub-study of the MRC National Survey of Health and Development, Insight 46, at age 69–71[1]. *Eligibility for recruitment was considered if participants met the criteria of having a specific set of life course data available (outlined below, in our protocol paper) and previously indicated they may be willing to attend a London-based clinic[1].

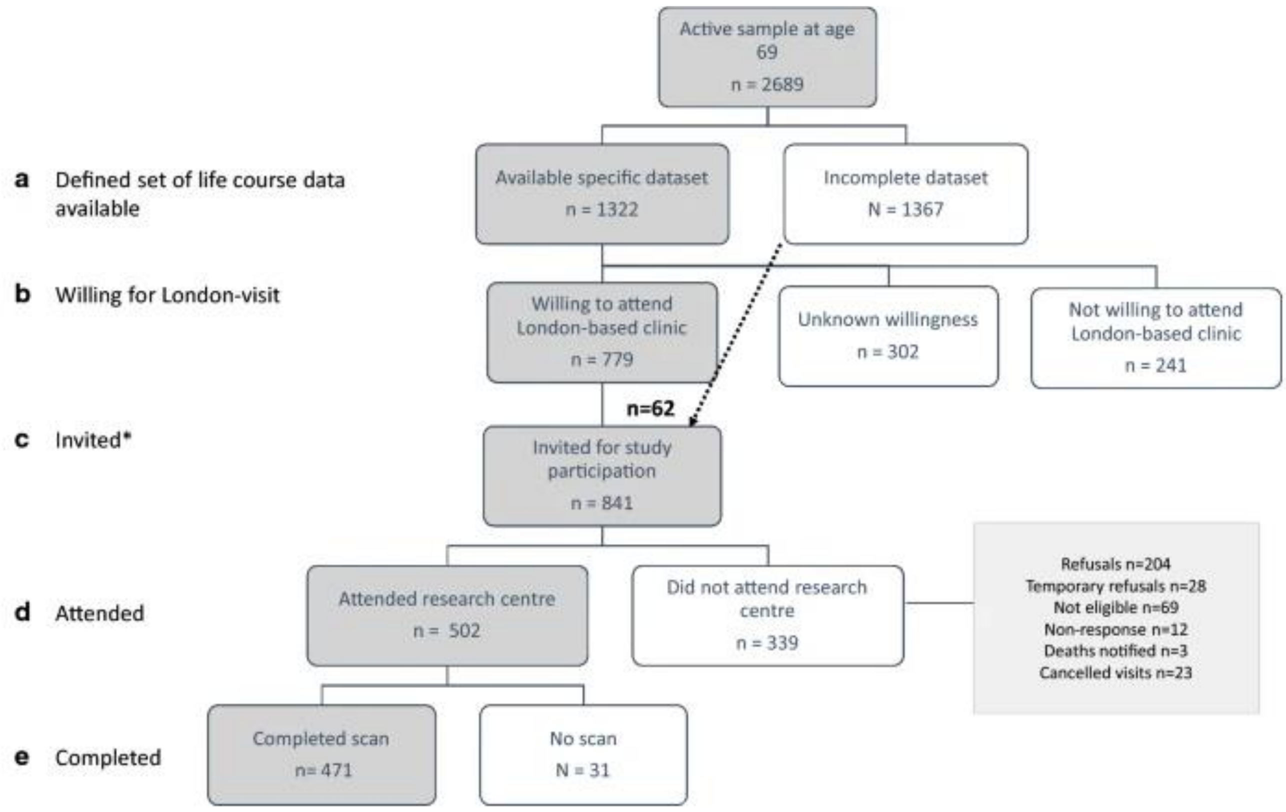

Original criteria of set of life course data available for Insight 46 eligibility *. Replicated from our protocol paper: [2]. When recruitment was underway the criteria were relaxed to remove the requirement for a measure of lung function, smoking or physical exercise (n=62).

Attendance at a clinic visit at age 60–64.
Parental socioeconomic position: at least one indicator of occupational social class or education.

Cognition: memory and processing speed from the 60–64 year collection AND at least one set of measures at either ages 8, 11 or 15.

Early physical growth trajectories: birth weight and at least one measure of height and weight at ages 4–15.

Educational attainment: highest qualification by age 26.

Mental health: teacher ratings of behaviour and temperament at ages 13 or 15, and at least one measure of affective symptoms at ages 36, 43, 53 or 60–64.

Blood pressure, lung function, adult height and weight: at least one measure of each at ages 36, 43, 53 or 60–64.

Health behaviours: at least one measure of smoking and physical exercise at ages 36, 43, 53 or 60–64.

Blood: either age 53 or 60–64 samples.

Supplementary Table 1: Descriptive characteristics of the analytical and wider NSHD sample. *SD, standard deviation, IQR=interquartile range, APOE-e4: apolipoprotein E.*

		**Analytical Sample (n=471)**	**Whole NSHD (n=2357)**
**Demographics**			
Sex	Female (n, %)	230 (49%)	1,228 (51%)
APOE-ε4	APOE ε4 non-carriers (n, %)	308 (70%)	1,415 (69.9%)
	APOE ε4 carriers (n, %)	130 (30%)	608 (30.1%)
Childhood social class	Manual (n, %)	198 (42%)	1,041 (46.5%)
	Non-manual (n, %)	269 (58%)	1,200 (53.5%)
Educational attainment	No qualifications (n, %)	74 (16%)	733 (32.6%)
	Up to age 16 (n, %)	142 (30%)	641 (28.5%)
	Up to age 17 or higher (n, %)	255 (54%)	875 (38.9%)
**Leisure time physical activity (LTPA) patterns**			
Active at age 36	None (n, %)	118 (27.2%)	717 (33.7%)
	Active ≥1times/month (n, %)	316 (72.8%)	1,410 (66.3%)
Active at age 43	None (n, %)	176 (38.8%)	1,066 (48.5%)
	Active ≥1times/month (n, %)	278 (61.2%)	1,133 (51.5%)
Active at age 53	None (n, %)	151 (32.6%)	963 (44.4%)
	Active ≥1times/month (n, %)	312 (67.4%)	1,208 (55.6%)
Active at age 60	None (n, %)	240 (51.5%)	1,199 (62.5%)
	Active ≥1times/month (n, %)	226 (48.5%)	719 (37.5%)
Active at age 69	None (n, %)	212 (45.7%)	1,412 (60.7%)
	Active ≥1times/month (n, %)	252 (54.3%)	915 (39.3%)
Life course patterns of LTPA (≥1 times per month) across different time periods	Never active (n, %)	30 (7%)	316 (13.9%)
	Active before 50's only (≤43 years)	54 (12%)	417 (18.3%)
	Active from 50's onwards (≥53 years)	51 (11%)	258 (11.3%)
	Always active (up to age 69 years)	329 (70%)	1,289 (56.5%)
**Cognitive function**			
Childhood cognition, age 8	Standardised mean (SD)	0.4 (0.7)	0.1 (0.8)
Cognitive state, age 69	Total score out of 100 (SD)	93 (4.6)	92 (5.7)

Supplementary material II: Is there a direct relationship between LTPA and brain health

Supplementary Table 2: The relationship between life course leisure time physical activity (LTPA) patterns with AD-related neurodegeneration measures at age 70.

	Aβ status			Hippocampal volume			Brain volume			CT AD-regions		
	OR	*p*	*CI 95%*	*Std ß*	*p*	*CI 95%*	*Std ß*	*p*	*CI 95%*	*Std ß*	*p*	*CI 95%*
Never active	Reference			Reference			Reference			Reference		
Active before 50’s only (≤43 years)	1.25	0.71	0.38-4.16	**0.56**	**0.02**	**0.09-1.02**	0.15	0.53	-0.31-0.60	0.10	0.66	-0.36-0.57
Active from 50’s onwards only (≥53 years)	0.79	0.72	0.22-2.84	0.32	0.19	-0.15-0.79	0.40	0.09	-0.07-0.86	0.34	0.16	-0.13-0.81
Always active	1.06	0.92	0.38-2.96	**0.41**	**0.03**	**0.01-0.80**	0.33	0.1	-0.06-0.71	0.27	0.18	-0.13-0.66
Interactions with:												
Sex	0.78			0.87			0.87			0.42		
APOE-e4	0.30			0.47			0.37			0.48		
WMHV tertiles	0.80			0.75			0.40			0.56		

Odds ratio (OR) with 95% confidence intervals are presented from logistic regression (Aβ status) and standardised coefficients (*Std ß* difference in standard deviation) and 95% confidence intervals are presented from linear regression models (hippocampal volume, HV; total brain volume, BV; Cortical thickness in Alzheimer’s-disease regions, CT AD): which assess brain health metrics at age 70 between those who were I) never active across adulthood; vs those who were active (participated in LTPA ≥1 times per month) II) before 50’s only (age≤43); III) from 50’s onwards only (age ≥53); IV) always active throughout adulthood. All models adjusted for sex, scan age, childhood cognition, childhood social class, education; and total intracranial volume (for HV, BV).

Associations between leisure time physical activity (LTPA) patterns and brain health measures at age 70, with extra adjustments.

Aim:
To re-run main analyses, adjusting for manual labour, history of poor mental health, and having poor general health (including longstanding illness, chronic pain, and pain that limits daily activity).

Methods:
Manual labour: Adulthood manual worker status was recorded from the participants most skilled occupation on record up to the age of 60 and categorised according to the Registrar General's classification and dichotomized into 'partly skilled or skilled manual work' and 'Skilled non-manual, intermediate or professional work'. There were 71 (15%) of participants who were considered to be 'partly skilled or skilled manual workers'.

To investigate how poor health may confound some of these relationships, we re-ran the main analyses adjusting for a number of poor health indices up to age 69.

Mental health: History of having a mental health affective disorder in adulthood was recorded as ever (yes/no) meeting the validated thresholds across assessments at ages 36, 43, 53, 60 and 69. There were 118 (34%) of participants who were recorded as ever having history of poor mental health.

General health and illness: At age 69, self-reported general health was rated on the postal questionnaire using a five point Likert-scale from excellent to poor, which was further categorised into 0) Excellent, very good and good (93%); and 1) fair or poor health (7%). Participants were additionally asked whether there was a longstanding illness, no (45%) or yes (55%). At age 69, participants were asked about whether they had pain that limited their daily activities (no/yes a little/yes a lot) which was further categorized into 0) No, yes a little (92%) 1) Yes a lot (8%). Participants who reported any ache or pain which had lasted for one day or longer (excluding pain because of a feverish illness) were asked whether they had experienced this pain for at least 3 months, and then to shade the location using a four-view body manikin. Chronic pain (35%) was also defined according to American College of Rheumatology criteria for fibromyalgia as pain present for 3 months or longer, above and below the waist; bilateral; and in the axial skeleton. Participants who reported chronic pain but did not meet the CWP definition were classified as having chronic regional pain (CRP)[3]. A general measure of poor physical health and pain was derived using all of these metrics which included either of the following: 1) fair or poor self-rated health 2) longstanding illness 3) experiencing a lot of pain that limited daily activities and 4) widespread or regional chronic pain. There were 298 (65%) of participants who were recorded as having poor physical health.

Results:
Supplementary Figure 2 shows the regression coefficients. When manual work (orange) was incorporated into the models, the results remained largely similar. When history of mental health (green) or having poor physical health (red) were additionally incorporated into the

models, the relationships between leisure time physical activity and hippocampal volume strengthened.

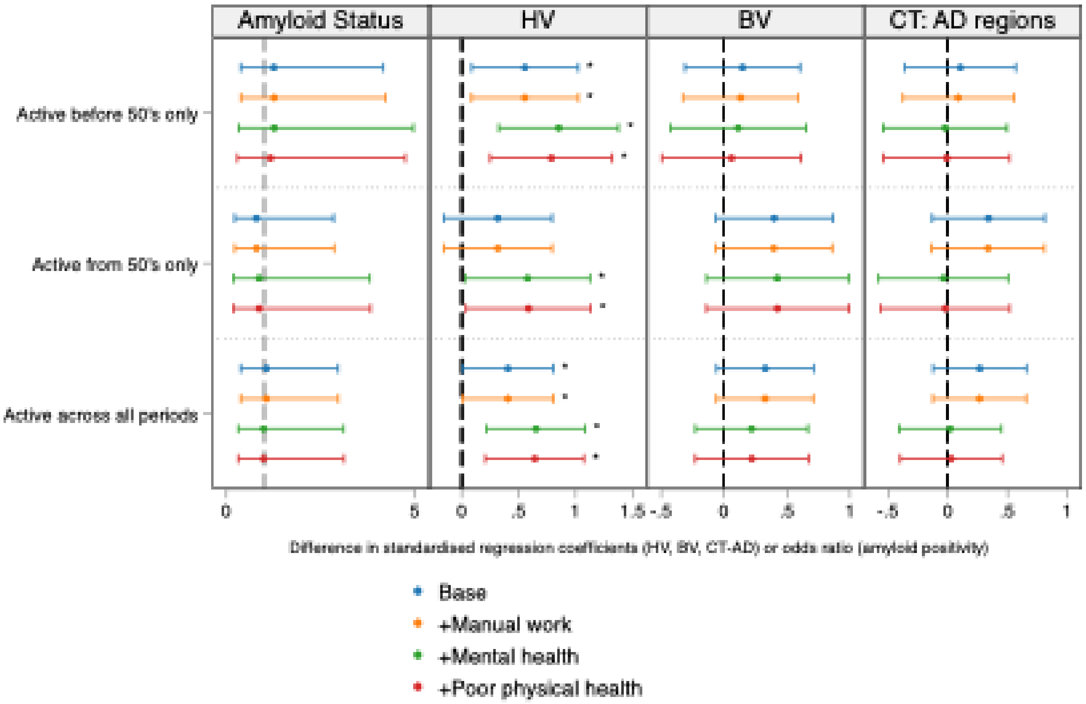

Supplementary Figure 2: Associations between leisure time physical activity (LTPA) patterns and brain health measures at age 70, with extra adjustments.
Odds ratio with 95% confidence intervals are presented from logistic regression (Aβ status: n=459) and standardised coefficients and 95% confidence intervals are presented from linear regression models (hippocampal volume, HV; total brain volume, BV; Cortical thickness in Alzheimer's-disease regions, CT AD; n=468): which assess brain health metrics at age 70 between those who were I) never active across adulthood; those who were active (participated in LTPA ≥1 times per month) II) before 50's only (age≤43); III) from 50's onwards only (age ≥53); IV) active throughout adulthood.

The first model (blue) was the base model, adjusting for sex, scan age, childhood cognition, childhood social class, education; and total intracranial volume (for HV, BV).
The second model (orange) additionally adjusted for adulthood manual work.
The third model (green) additionally adjusted for history of ever having a mental health disorder.
The fourth mode (red) additionally adjusted for having poor physical health and pain (self-rating health as poor, having longstanding illness, having chronic pain, and having pain that severely limits daily activity).

Supplementary material III: The indirect relationship between LTPA, brain health and cognition, with adjustments

Aim:
To re-run main analyses, adjusting for manual labour, history of poor mental health, and having poor general health (including longstanding illness, chronic pain, and pain that limits daily activity) (see supplementary material II for definitions).

Results

When manual work was incorporated into the model the relationships remained similar.

Results remained largely similar with adjustment for manual work (Supplementary Table 3). The relationships between brain health (amyloid status, hippocampal volume and CT-AD) with cognition in the inactive group strengthened with adjustment for poor mental or physical health (Supplementary Table 4) (e.g. CT-AD and cognition in base model: 0.31 [0.13,0.50] p<0.01 and fully adjusted model: 0.56 [0.34,0.78] p<0.01).

Supplementary Table 3: The relationship between brain health measures and cognition function at age 70 (Preclinical Alzheimer's cognitive composite, PACC), by life course leisure time physical activity (LTPA) patterns, with adjustment for manual work.

	ß	*p*	*CI 95%*	*P-value for overall modification effect by LTPA*
**Aβ status and PACC**				
By LTPA patterns:				**<0.01**
Never active	**-1.60**	**0.01**	**-2.20--1.00**	
Active before 50's only (≤43 years)	-0.09	0.66	-0.50-0.32	
Active from 50's onwards only (≥53 years)	-0.32	0.2	-0.81-0.17	
Always active	-0.17	0.05	-0.34-0.00	
**Hippocampal volume and PACC**				
By LTPA patterns:				**<0.01**
Never active	**0.44**	**0.01**	**0.26-0.62**	
Active before 50's only (≤43 years)	0.10	0.22	-0.06-0.25	
Active from 50's onwards only (≥53 years)	0.02	0.81	-0.15-0.19	
Always active	0.01	0.86	-0.06-0.08	
**Brain volume and PACC**				
By LTPA patterns:				**<0.01**
Never active	**0.27**	**0.01**	**0.07-0.46**	
Active before 50's only (≤43 years)	**0.26**	**<0.01**	**0.10-0.43**	
Active from 50's onwards only (≥53 years)	0.10	0.25	-0.07-0.27	
Always active	-0.01	0.85	-0.08-0.06	
**Cortical thickness in AD-regions and PACC**				**0.04**
By LTPA patterns:				
Never active	**0.32**	**<0.01**	**0.14-0.51**	
Active before 50's only (≤43 years)	0.04	0.68	-0.15-0.23	
Active from 50's onwards only (≥53 years)	0.17	0.07	-0.01-0.35	
Always active	0.07	0.08	-0.02-0.14	

Coefficients and 95% confidence intervals are presented from linear regression models which assess the relationship between brain health (Aβ status; standardised hippocampal volume; standardised total brain volume; standardised cortical thickness in Alzheimer's-disease regions) and cognition at age 70 (Preclinical Alzheimer's cognitive composite, PACC). The overall modification effect by LTPA column indicates the p-value for LTPA on brain health and PACC scores. Coefficients 'by LTPA patterns' show the marginal effects of the relationship between brain health and PACC, by LTPA patterns (I) never active across adulthood; those who were active (participated in LTPA ≥1 times per month) in II) before 50's only (age≤43); III) from 50's onwards only (age ≥53); IV) always active throughout adulthood). All models adjusted for sex, scan age, childhood cognition, childhood social class, education; and total intracranial volume (for HV, BV) and adult manual working class.

Supplementary Table 4: The relationship between brain health measures and cognition function at age 70 (Preclinical Alzheimer's cognitive composite, PACC), by life course leisure time physical activity (LTPA) patterns, with adjustment for mental and physical poor health.

	ß	*p*	*CI 95%*	*P-value for overall modification effect by LTPA*
**Aβ status and PACC**				**<0.01**
With interaction by LTPA patterns:				
Never active	-1.85	**0.01**	-2.53--1.18	
Active before 50's only (≤43 years)	0.14	0.58	-0.36-0.65	
Active from 50's onwards only (≥53 years)	-0.54	0.07	-1.12-0.05	
Always active	-0.12	0.25	-0.31-0.08	
**Hippocampal volume and PACC**				**<0.01**
By LTPA patterns:				
Never active	**0.58**	**<0.01**	**0.34-0.83**	
Active before 50's only (≤43 years)	0.08	0.46	-0.13-0.28	
Active from 50's onwards only (≥53 years)	0.13	0.28	-0.11-0.37	
Always active	-0.03	0.43	-0.11-0.05	
**Brain volume and PACC**				**<0.01**
By LTPA patterns:				
Never active	**0.27**	**0.02**	**0.04-0.49**	
Active before 50's only (≤43 years)	**0.33**	**<0.01**	**0.14-0.51**	
Active from 50's onwards only (≥53 years)	0.16	0.13	-0.05-0.37	
Always active	0.00	0.94	-0.08-0.08	
**Cortical thickness in AD-regions and PACC**				<**0.01**
By LTPA patterns:				
Never active	**0.56**	**<0.01**	**0.34-0.78**	
Active before 50's only (≤43 years)	0.10	0.45	-0.16-0.35	
Active from 50's onwards only (≥53 years)	0.21	0.06	-0.01-0.43	
Always active	0.02	0.56	-0.06-0.10	

Coefficients and 95% confidence intervals are presented from linear regression models which assess the relationship between brain health (Aβ status; standardised hippocampal volume; standardised total brain volume; standardised cortical thickness in Alzheimer's-disease regions) and cognition at age 70 (Preclinical Alzheimer's cognitive composite, PACC). The overall modification effect by LTPA column indicates the p-value for LTPA on brain health and PACC scores. Coefficients 'by LTPA patterns' show the marginal effects of the relationship between brain health and PACC, by LTPA patterns (I) never active across adulthood; those who were active (participated in LTPA ≥1 times per month) in II) before 50's only (age≤43); III) from 50's onwards only (age ≥53); IV) always active throughout adulthood). All models adjusted for sex, scan age, childhood cognition, childhood social class, education; and total intracranial volume (for HV, BV), manual working class and history of ever having a mental health disorder, and having poor physical health and pain (self-rating health as poor, having longstanding illness, having chronic pain, and having pain that severely limits daily activity).

Supplementary Table 5: Sex-stratified models of the marginal relationship between A) Aβ status and cognition, B) cortical thickness in AD regions (CT-AD) and cognition, by leisure time physical activity (LTPA) patterns. Linear regression models assessing the relationship between each brain health measure of interest and outcome of interest at age 70 (max n=468), adjusting for scan age, childhood cognition, childhood social class, education; and with interaction terms between sex, LTPA patterns and relevant exposure imaging metrics of interest. LTPA patterns were compared between those who were I) never active across adulthood; those who were active (participated in LTPA ≥1 times per month) in II) before 50's only (age≤43); III) from 50's onwards only (age ≥53); IV) always active throughout adulthood. Std=standardized. SUVR=global standardized uptake value ratios. Wald tests revealed sex*LTPA*imaging metric interactions were all p<0.01.

	**Men**			**Women**		
	Relationship between brain health and cognition			Relationship between brain health and cognition		
	ß	*p*	*CI 95%*	*ß*	*p*	*CI 95%*
**A) Aβ status and PACC**	-0.25	<0.01	-0.46--0.05	-0.23	0.04	-0.46--0.01
By LTPA patterns:	-	-	-	-	-	-
Never active	-0.67	0.15	-1.58-0.24	-2.49	**<0.01**	-3.29--1.69
Active before 50's only (≤43 years)	-0.10	0.72	-0.62-0.42	0.01	0.98	-0.65-0.67
Active from 50's onwards only (≥53 years)	-0.3	0.38	-0.96-0.36	-0.45	0.23	-1.18-0.29
Always active	-0.29	0.41	-0.52--0.05	-0.02	0.91	-0.27-0.24
**B) Cortical thickness AD-regions and PACC**	0.06	0.10	-0.02-0.15	0.13	**<0.01**	0.05-0.21
By LTPA patterns:	-	-	-	-	-	-
Never active	0.01	0.94	-0.22-0.24	0.88	**<0.01**	0.58-1.18
Active before 50's only (≤43 years)	-0.02	0.86	-0.28-0.23	0.12	0.41	-0.17-0.41
Active from 50's onwards only (≥53 years)	0.11	0.43	-0.16-0.38	0.18	0.15	-0.06-0.43
Always active	0.07	0.20	-0.04-0.17	0.07	0.15	-0.02-0.16

Supplementary Figure 3: Marginal effects for sex and leisure time physical activity (LTPA) patterns for the relationship between brain health and cognition at age 70, removing females with the poorest cognitive performance. The data points represent marginal effects by sex of the relationship between the exposure and outcome, derived from linear regression models assessing the relationship between each brain health measure of interest and outcome of interest at age 70 (max n=468), adjusting for scan age, childhood cognition, childhood social class, education; and with interaction terms between sex, LTPA patterns and relevant exposure imaging metric of interest. LTPA patterns were compared between those who were I) never active across adulthood; those who were active (participated in LTPA ≥1 times per month) in II) before 50's only (age≤43); III) from 50's onwards only (age ≥53); IV) always active throughout adulthood. Std=standardized. SUVR=global standardized uptake value ratios.

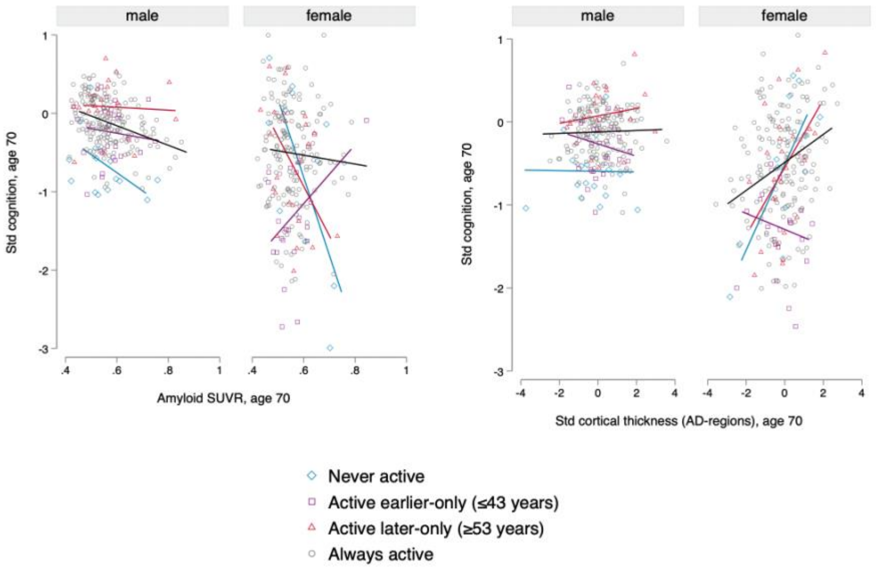

Aim and methods
To re-run sex-stratified analyses between LTPA, brain health (amyloid, cortical thickness) and cognition, removing the two women with the poorest cognitive function (below -3 SD on the PACC).

Results

- The LTPA*sex*amyloid interaction changes from p<0.01 to p=0.3, but the coefficients for inactive women remain significant (b=-0.96, p=0.04)
- The LTPA*sex*CT-AD interaction changes from p<0.01 to p=0.6 and the coefficient for inactive women is attenuated (b=-0.12, p=0.21)

Discussion
The relationship between brain health and cognition attenuated when two women with the poorest cognitive function were removed, suggesting that this relationship is largely driven by inactive women who have the poorest cognitive function and likely already have early stages of AD.

References

1. James SN, Lane CA, Parker TD, Lu K, Collins JD, Murray-Smith H, et al. Recruitment and participation patterns of a birth cohort neuroscience sub-study: Insight 46. BMC Research Notes.

2. Lane CA, Parker TD, Cash DM, Macpherson K, Donnachie E, Murray-Smith H, et al. Study protocol: Insight 46 – a neuroscience sub-study of the MRC National Survey of Health and Development. BMC Neurology. 2017 Dec 18;17(1):75.

3. Kuh D, Wong A, Shah I, Moore A, Popham M, Curran P, et al. The MRC National Survey of Health and Development reaches age 70: maintaining participation at older ages in a birth cohort study. European Journal of Epidemiology. 2016 Nov;31(11):1135–47.
